# Primary Cutaneous Histoplasmosis in a Human Immunodeficiency Virus (HIV) Seronegative Patient

**DOI:** 10.7759/cureus.113919

**Published:** 2026-08-04

**Authors:** Lyuva A Ramirez-Devars, Bianka Duoaney Barajas-Mendez, Roxana Torres-Sánchez, Aimee Nuñez-Gilabert, Enrique Alessio Garibo-Luna, Alexis Rivas-Antonio

**Affiliations:** 1 Department of Dermatology, Centro Médico Naval, Mexico City, MEX; 2 Department of Dermatology, Universidad Nacional Autónoma de México (UNAM), Mexico City, MEX; 3 Department of Internal Medicine, Instituto Mexicano del Seguro Social (IMSS), Mexico City, MEX; 4 Department of Internal Medicine, Centro Médico Naval, Mexico City, MEX; 5 Department of Internal Medicine, Universidad del Ejército y Fuerza Aérea Mexicanos (UDEFA), Mexico City, MEX

**Keywords:** dermatology, hiv seronegative, infectious disease, infectology, mycosis

## Abstract

Histoplasmosis is caused by *Histoplasma capsulatum.* Primary cutaneous histoplasmosis is an exceptionally rare entity. While historically described in immunosuppressed patients, its prevalence in immunocompetent hosts in Latin America is rising, often misdiagnosed due to highly polymorphic, non-specific lesions like papules, plaques, nodules, or ulcers. We present a clinical case of a human immunodeficiency virus (HIV)‑negative, otherwise immunocompetent patient with primary cutaneous histoplasmosis confined to the skin, diagnosed by periodic acid-Schiff (PAS) and Gomori-Grocott staining in the absence of fungal culture and successfully treated with prolonged itraconazole.

## Introduction

Histoplasmosis is an infection caused by the dimorphic fungus *Histoplasma capsulatum*, which grows at temperatures between 25°C and 28°C, developing microconidia and macroconidia. The pathogen is capable of invading neutrophils within the first 24 hours of infection and induces cell-mediated immunity approximately 21 days post-exposure [1]. It is frequently found in bird droppings, bat guano, and contaminated soil [2]; consequently, the primary risk factors involve exposure in immunosuppressed patients [3].

Histoplasmosis presents with a broad spectrum of clinical manifestations, and its primary route of infection is respiratory by the inhalation of microconidia [4]. Cutaneous histoplasmosis occurs in 4-11% of cases and is caused by hematogenous dissemination from a pulmonary focus in 80% of patients with untreated systemic histoplasmosis [5]. Conversely, primary cutaneous histoplasmosis, defined as infection limited to the skin due to direct inoculation rather than hematogenous dissemination, is rare, affecting less than 1% of patients with histoplasmosis. Its main risk factors include chronic occupational exposure or activities involving the disruption of soil contaminated with *H. capsulatum *in immunosuppressed individuals [2,5]. Some reports indicate a noteworthy prevalence of cutaneous histoplasmosis among immunocompetent patients in Latin America, which is believed to be secondary to diagnostic delays [4]. In immunocompetent hosts, this entity is extremely rare and remains asymptomatic in the majority of cases [6].

The cutaneous lesions caused by *H. capsulatum *are highly polymorphic and non-specific. It may present as painless ulcers associated with regional lymphadenopathy that develop over weeks or months. Lesions can be mutilating and include erythematous papules, plaques with or without a central crust, pustules, and nodules and may even mimic molluscum contagiosum, involving the extremities, trunk, and mucous membranes [4].

It is recommended that diagnosis be performed via histopathological examination of the lesions using periodic acid-Schiff (PAS) and Gomori-Grocott methenamine silver stains, which reveal spore-like fungal structures stained red and dark brown, respectively. The gold standard for confirmation is fungal culture of the lesions; however, this process typically takes 3-6 weeks [4,5].

The treatment of primary cutaneous histoplasmosis in immunocompetent patients consists of itraconazole (200 mg orally) three times daily for three days, followed by twice daily for 12-24 months, given that this entity exhibits high relapse rates with shorter regimens [1].

## Case presentation

**Figure 1:**
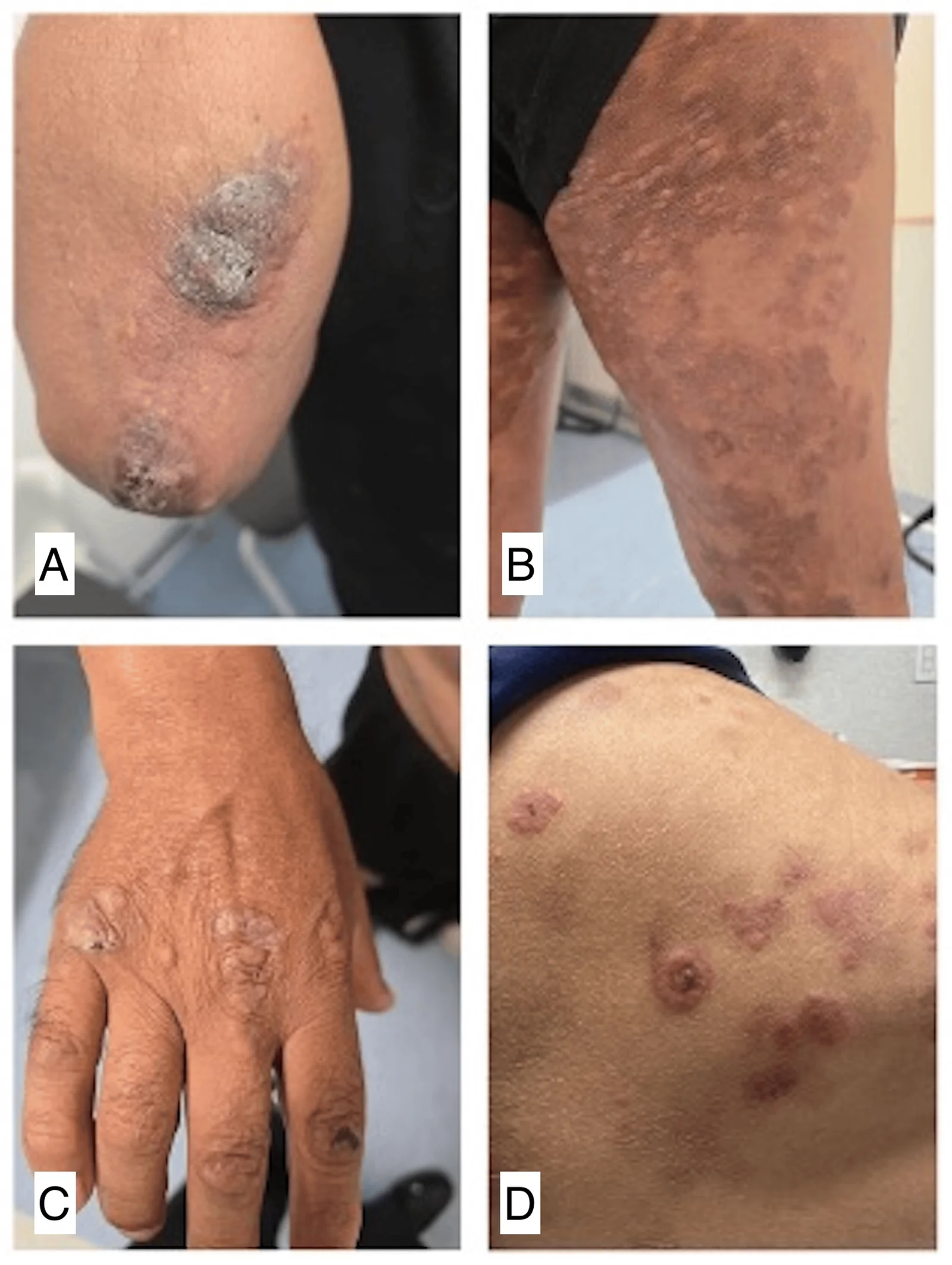
Disseminated dermatosis characterized by multiple erythematous nodules with overlying surface crusting (A) Nodules 1 and 2 cm in diameter located on the right elbow, with an attached thick, grayish scale. (B) Plaques made up of multiple 5-mm-diameter nodules on a light brown macule. (C) Skin-colored nodules 5 mm in size on the dorsum of the right hand. (D) Multiple reddish, umbilicated nodules 1 cm in diameter on the posterior thorax.

We report the case of a 69-year-old male bricklayer with a significant smoking history from ages 17 to 50 (15 cigarettes/day) and a current diagnosis of systemic arterial hypertension managed with enalapril (10 mg every 24 hours). The patient presented with a dermatosis affecting the upper and lower limbs, with a predominance on the trunk. The eruption was characterized by indurated erythematous-squamous plaques, focal exulcerations, and scattered erythematous nodules (Figure 1), accompanied by mild pruritus (visual analog scale: 3/10) of one year's evolution.

Dermoscopic evaluation revealed silvery scales and punctate vessels, some displaying active bleeding. Based on an initial clinical suspicion of plaque psoriasis, topical therapy with calcipotriol/betamethasone was initiated, and workup for systemic therapy was underway. The patient returned for reevaluation after two months of treatment, presenting with a severe exacerbation of the dermatosis. This was characterized by an increased number of erythematous nodules, the emergence of serous vesicles and bullae, and some plaques exhibiting central atrophy with an active-appearing border, accompanied by burning pain (5/10).

**Figure 2:**
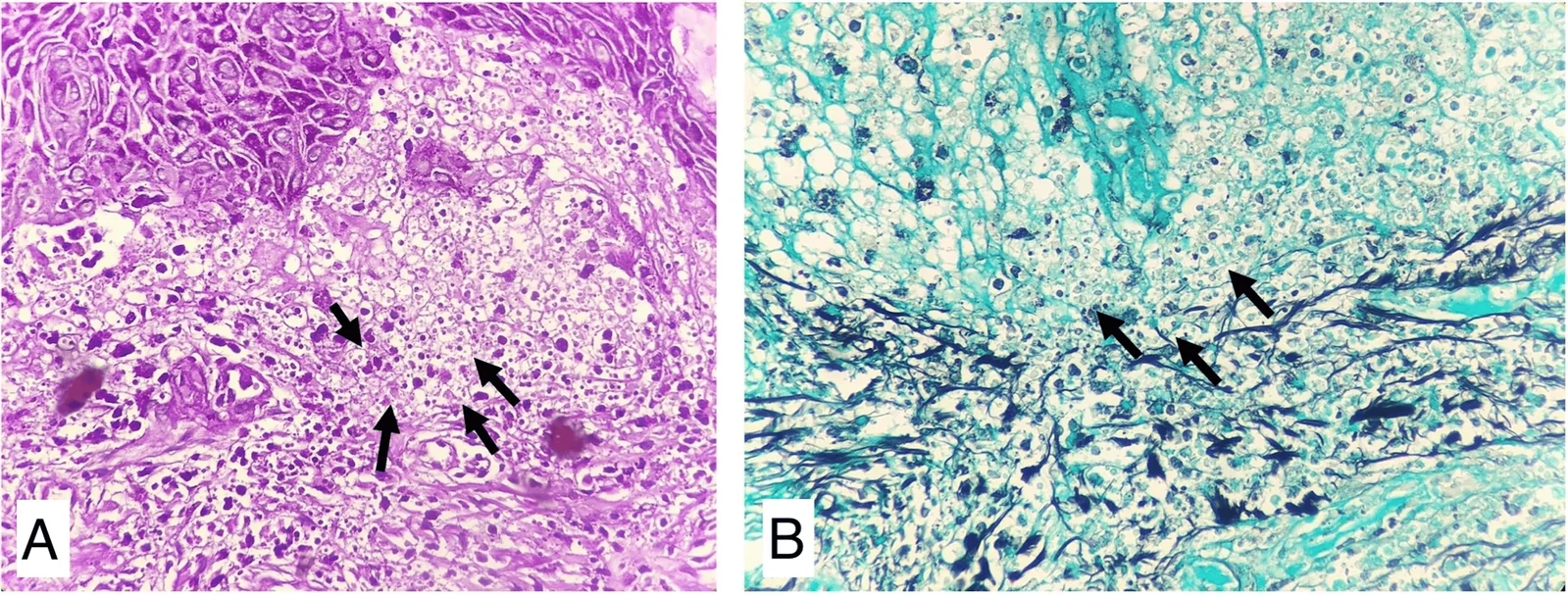
Periodic acid-Schiff and Gomori-Grocott stains (magnification 40×) (A) In the periodic acid-Schiff stain, the arrows indicate multinucleated giant cells and multiple oval-shaped, magenta-colored yeast-like structures. (B) In the Gomori-Grocott stain, the arrows indicate multiple oval-shaped, brown structures, microorganisms morphologically consistent with* Histoplasma *sp.

A skin biopsy was performed under the differential diagnosis of cutaneous lymphoma versus an infectious dermatosis. Histopathological analysis with PAS staining revealed multinucleated giant cells and multiple oval, magenta-colored yeast-like structures. The Gomori-Grocott silver stain demonstrated numerous brown oval structures, displaying a morphology highly consistent with *Histoplasma *spp. (Figure 2).

**Figure 3:**
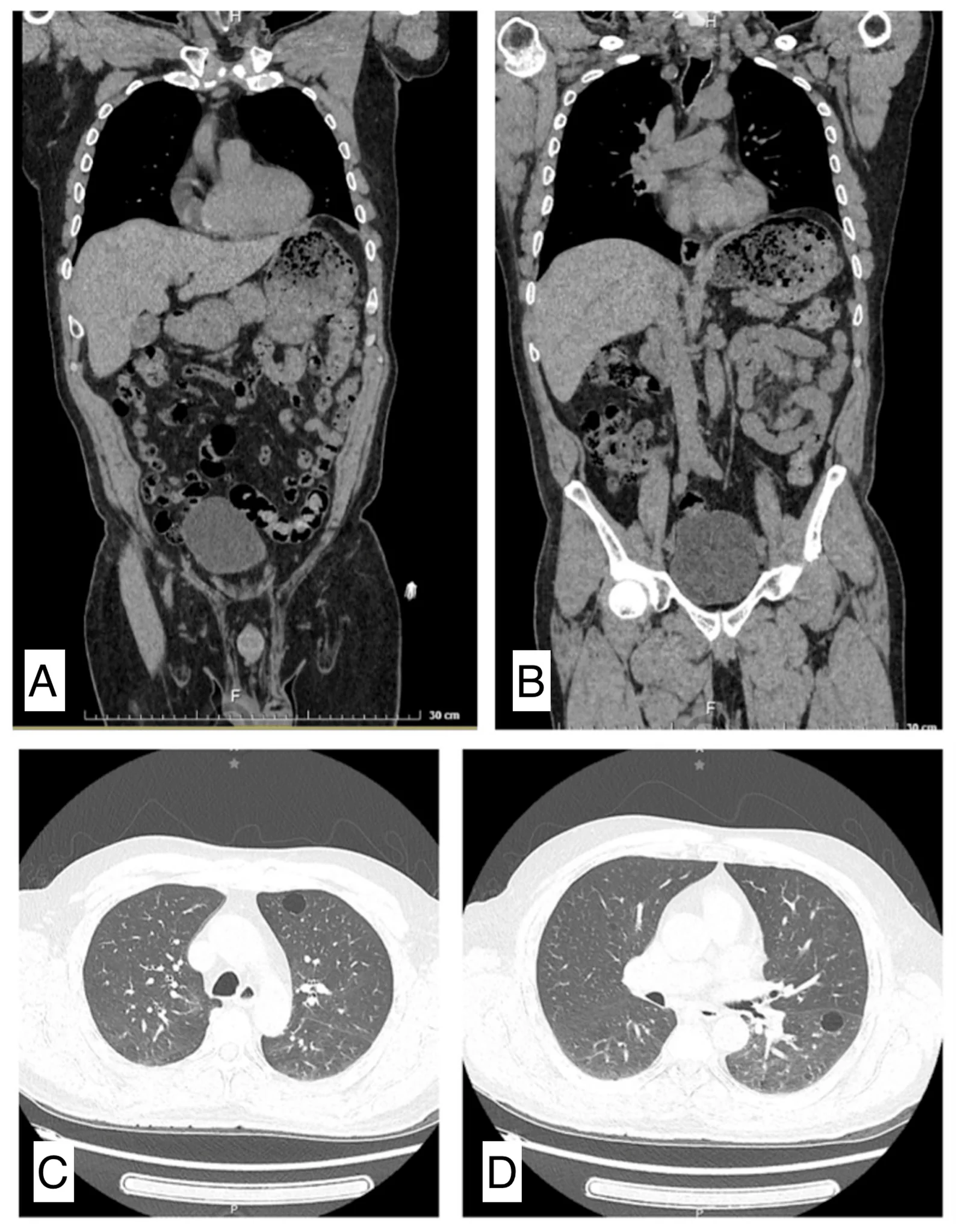
Non-contrast thoracoabdominopelvic computed tomography (A, B) Coronal reconstructions demonstrating no thoracic or abdominal findings suggestive of disseminated histoplasmosis. (C, D) Lung window images showing mild thin-walled pulmonary cysts without pulmonary nodules, consolidations, cavitary lesions, or other computed tomography findings suggestive of pulmonary histoplasmosis.

The patient was hospitalized to screen for disseminated histoplasmosis. A high‑resolution computed tomography (HRCT) scan of the chest showed no nodules, consolidations, cavitary lesions, or other findings suggestive of pulmonary histoplasmosis. The study was extended to the abdomen and pelvis to rule out involvement of other organs (Figure 3). Taken together with the absence of systemic symptoms and normal examination, this supported infection confined to the skin rather than systemic dissemination. In addition, TORCH (toxoplasmosis, other infections, rubella, cytomegalovirus, and herpes simplex virus) panel, QuantiFERON-TB Gold, and viral serologies for hepatitis and human immunodeficiency virus (HIV) were all negative. Management for primary cutaneous histoplasmosis was initiated with oral itraconazole (200 mg orally) three times daily for three days, followed by twice daily for 12 months. The patient demonstrated significant clinical improvement after three months of therapy.

## Discussion

Primary cutaneous histoplasmosis is an exceedingly rare clinical entity. Dao et al. reported that only 1% of histoplasmosis lesions are isolated strictly to the skin, with pulmonary involvement being the most common presentation [2]. In contrast to that series, our patient presented as a man with a completely negative pulmonary workup and infection strictly confined to the cutaneous tissue. Furthermore, Sahu et al. reported that the majority of patients diagnosed with histoplasmosis were HIV-positive [6], which stands in contrast to the HIV-negative status of our patient.

Batista et al. emphasize fungal culture as a cornerstone of the diagnostic workup. However, isolating the pathogen in primary cutaneous histoplasmosis can be challenging; the main disadvantages of culture are its low sensitivity in certain samples and a prolonged incubation period of up to six weeks [5]. In our case, access to a mycology laboratory capable of prolonged culture was limited, making fungal culture logistically unfeasible. Diagnosis therefore relied on PAS and Gomori‑Grocott stains, which demonstrated numerous intracellular oval yeast‑like structures with narrow‑based budding, morphologically consistent with *Histoplasma *spp. Also, it is of clinical relevance that despite being a localized infection without systemic organ involvement, it mandates prolonged therapy (up to one year) with oral itraconazole, reserving intravenous amphotericin B for severe, disseminated cases or HIV-positive individuals [2].

## Conclusions

Primary cutaneous histoplasmosis remains an exceptional entity, but should be considered in chronic, atypical dermatoses even in immunocompetent patients without evidence of systemic involvement. This case highlights the clinical imperative of including *H. capsulatum *within the differential diagnosis of chronic dermatoses with an atypical course, especially in individuals with occupational exposure to potentially contaminated soil. The non-specific clinical presentation and its morphological overlap with other inflammatory or neoplastic dermatoses can significantly delay diagnosis, as previously documented in the literature.

Histopathological confirmation using special stains (PAS and Gomori-Grocott) proved essential in this case, particularly given the unavailability of a fungal culture, whose long processing time represents a frequent real-world limitation. The absence of pulmonary involvement and negative HIV serology align with recent reports describing sporadic cases in immunocompetent patients, despite its low overall prevalence.

Prolonged treatment with itraconazole led to a favorable clinical outcome, consistent with current guideline recommendations aimed at preventing disease relapse. This case reinforces the need to maintain close clinical surveillance and to recognize that, although rare, primary cutaneous histoplasmosis can arise in patients without immunosuppression, demanding a timely diagnostic approach and appropriate antifungal management.
